# Supplementary material for: Changes to information in working memory depend on distinct removal operations
Source: Nat Commun. 2020 Dec 7;11:6239. doi: 10.1038/s41467-020-20085-4 (PMC7721711; doi:10.1038/s41467-020-20085-4)
Supplement: Supplementary file 1 — Supplementary Information [file 41467_2020_20085_MOESM1_ESM.pdf]

Supplementary Information

Changes to information in working memory depend on distinct removal operations

Kim et al.

Supplementary Figures

Supplementary Figure 1. Brain regions that show differential activation for suppress, clear, and maintain**.** A) Suppress vs. clear operation. Much of lateral prefrontal cortex exhibits greater activity for the suppress than the clear condition (shown in warm colors), consistent with the idea that suppression is an active and controlled process. In contrast, regions in posterior cortex that have been associated with distraction show more activity than the clear condition (shown in cool colors). B) Suppress vs. maintain operation. Greater activation is observed in the suppress than the maintain items in inferior frontal and opercular regions involved in cognitive control (shown in warm colors). In contrast, regions of the ventral visual stream show more activation for the maintain than suppress condition (shown in cool colors) consistent with the idea that specifics of items remain to a greater degree in the maintain than suppress condition. The color bar represents t-values. The cortical labels were adapted from Mindboggle-101 provided in FreeSurfer [1]. S/M/I: superior/middle/inferior, F/P/T: frontal/parietal/temporal, LO: lateral occipital, AC/PC: anterior/posterior cingulate, LH/RH: left/right hemisphere.

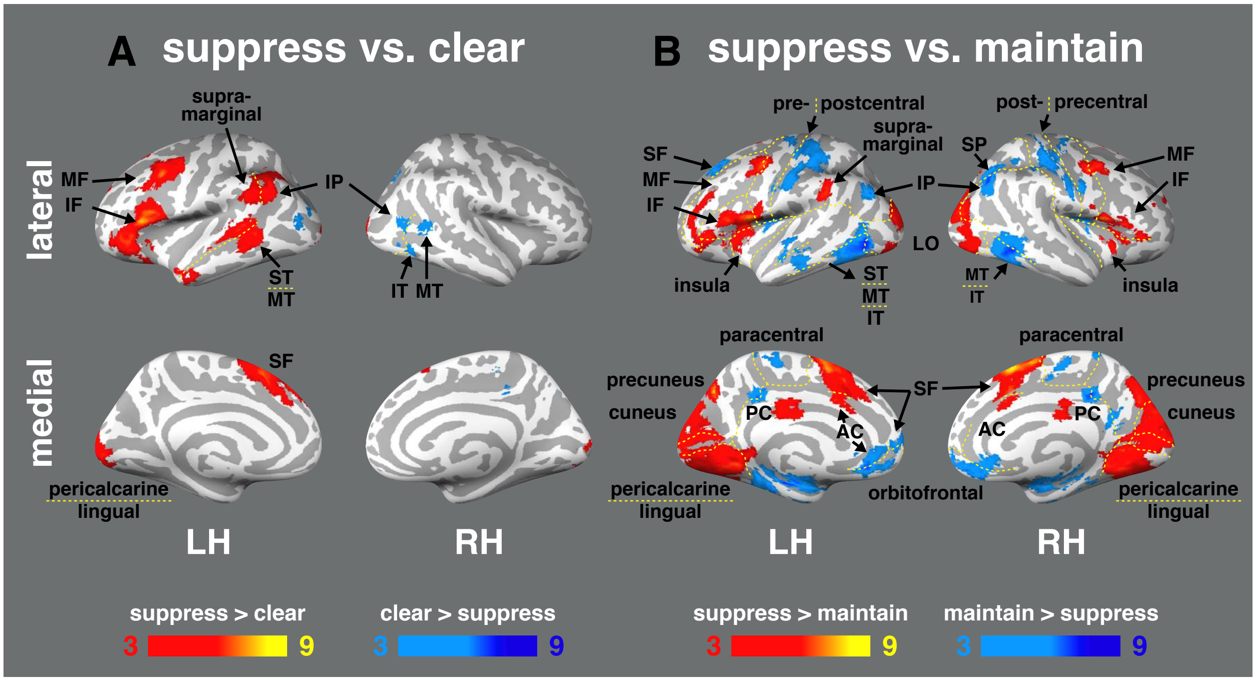

Supplementary Tables

Supplementary Table 1. Summary statistics of the WM operation classifiers. The classifier was trained with and tested on the central study data (k-fold leave-one-out cross-validation).

Within-subject classification

5-operation classifier	Individualized penalty				
	M±SEM	T(49)	P	D	CI
Maintain	0.43±0.02<br>0.71±0.02	10.27<br>12.83	8.28e-14<br>< 0.001	1.452<br>1.815	[0.18, 0.27]<br>[0.17, 0.24]
Replace (category)	0.33±0.01<br>0.67±0.01	14.71<br>15.5	< 0.001<br>< 0.001	2.08<br>2.192	[0.11, 0.14]<br>[0.15, 0.19]
Replace (subcategory)	0.34±0.01<br>0.69±0.01	15.14<br>17.76	< 0.001<br>< 0.001	2.142<br>2.512	[0.13, 0.16]<br>[0.16, 0.21]
Suppress	0.48±0.03<br>0.74±0.02	10.61<br>13.7	2.72e-14<br>< 0.001	1.5<br>1.937	[0.23, 0.33]<br>[0.21, 0.28]
Clear	0.51±0.03<br>0.77±0.02	11.97<br>16.52	3.33e-16<br>< 0.001	1.693<br>2.337	[0.26, 0.36]<br>[0.24, 0.31]

4-operation classifier — Individualized penalty | Global penalty (penalty = 50)

4-operation classifier	M±SEM		T(49)		P		D		CI	
Maintain	0.47±0.02<br>0.71±0.02	0.47±0.02<br>0.71±0.02	10.27<br>12.25	10.07<br>12.16	8.37e-14<br>1.11e-16	1.62e-13<br>2.22e-16	1.452<br>1.733	1.424<br>1.72	[0.18, 0.27]<br>[0.17, 0.24]	[0.18, 0.26]<br>[0.17, 0.24]
Replace (category)	0.53±0.02<br>0.77±0.02	0.53±0.02<br>0.77±0.02	15.55<br>17.48	15.06<br>17.63	< 0.001<br>< 0.001	< 0.001<br>< 0.001	2.2<br>2.472	2.13<br>2.492	[0.25, 0.32]<br>[0.23, 0.3]	[0.24, 0.32]<br>[0.24, 0.3]
Suppress	0.50±0.02<br>0.72±0.02	0.50±0.02<br>0.72±0.02	10.26<br>12.36	10.07<br>12.30	8.55e-14<br>1.11e-16	1.58e-13<br>1.11e-16	1.451<br>1.749	1.425<br>1.739	[0.2, 0.3]<br>[0.19, 0.26]	[0.2, 0.3]<br>[0.19, 0.26]
Clear	0.53±0.02<br>0.75±0.02	0.53±0.02<br>0.75±0.02	11.84<br>14.57	11.78<br>14.64	5.55e-16<br>< 0.001	6.66e-16<br>< 0.001	1.674<br>2.061	1.666<br>2.071	[0.24, 0.33]<br>[0.22, 0.28]	[0.23, 0.33]<br>[0.22, 0.28]

For each operation, the statistics for the classifier accuracy (row 1) and AUC (row 2) are summarized in the mean ± SEM, *t*-value (DOF = 49) of one-sample T-tests (two-sided) with chance level (1/the number of operations for accuracy, 0.5 for AUC), *p*-value, effect size (Cohen's *d*), and 95% confidence interval in order.

Between-subject classification (4-operation, anatomically aligned)

4-operation classifier	Global penalty (penalty = 50)				
	M±SEM	T(48)	P	D	CI
Maintain	0.39±0.01	10.88	1.51e-14	1.554	[0.12, 0.17]
Replace (category)	0.47±0.01	17.19	3.78e-22	2.456	[0.2, 0.25]
Suppress	0.34±0.01	6.4	6.2e-08	0.914	[0.06, 0.12]
Clear	0.4±0.02	8.24	9.44e-11	1.178	[0.11, 0.19]

For each operation, the statistics for the classifier accuracy are summarized in the mean ± SEM, *t*-value (DOF = 48) of one-sample T-tests (two-sided) with chance level = 0.25, *p*-value, effect size (Cohen's *d*), and 95% confidence interval in order.

Supplementary Table 2. Summary statistics of the WM representation classifiers. The classifier was first trained and tested on the localizer data (k-fold leave-one-out cross-validation). Then, the classifier was trained with the entire localizer data and tested on the central study data.

<table>
<tr><th rowspan="2">Category</th><th colspan="5">Individualized penalty | Global penalty (penalty = 50)</th></tr>
<tr><th>M±SEM</th><th>T(49)</th><th>P</th><th>D</th><th>CI</th></tr>
<tr><td rowspan="3">Face</td><td>0.84±0.01 | 0.85±0.01</td><td>34.62 | 35.11</td><td>< 0.001 | < 0.001</td><td>4.895 | 4.965</td><td>[0.48, 0.54] | [0.48, 0.54]</td></tr>
<tr><td>0.93±0.01 | 0.93±0.01</td><td>43.76 | 45.33</td><td>< 0.001 | < 0.001</td><td>6.189 | 6.411</td><td>[0.41, 0.45] | [0.42, 0.45]</td></tr>
<tr><td>0.85±0.01</td><td>40.38</td><td>< 0.001</td><td>5.711</td><td>[0.49, 0.55]</td></tr>
<tr><td rowspan="3">Fruit</td><td>0.75±0.02 | 0.75±0.02</td><td>27.02 | 26.74</td><td>< 0.001 | < 0.001</td><td>3.822 | 3.781</td><td>[0.39, 0.45] | [0.38, 0.45]</td></tr>
<tr><td>0.87±0.01 | 0.87±0.01</td><td>33.14 | 32.76</td><td>< 0.001 | < 0.001</td><td>4.687 | 4.632</td><td>[0.35, 0.4] | [0.35, 0.4]</td></tr>
<tr><td>0.71±0.01</td><td>28.16</td><td>< 0.001</td><td>3.983</td><td>[0.35, 0.4]</td></tr>
<tr><td rowspan="3">Scene</td><td>0.82±0.01 | 0.81±0.01</td><td>40.63 | 38.52</td><td>< 0.001 | < 0.001</td><td>5.746 | 5.447</td><td>[0.46, 0.51] | [0.46, 0.51]</td></tr>
<tr><td>0.92±0.01 | 0.92±0.01</td><td>51.75 | 49.97</td><td>< 0.001 | < 0.001</td><td>7.318 | 7.067</td><td>[0.4, 0.44] | [0.4, 0.44]</td></tr>
<tr><td>0.85±0.01</td><td>36.72</td><td>< 0.001</td><td>5.194</td><td>[0.49, 0.5]</td></tr>
<tr><th rowspan="2">Subcategory</th><th colspan="5">Individualized penalty | Global penalty (penalty = 50)</th></tr>
<tr><th>M±SEM</th><th>T(49)</th><th>P</th><th>D</th><th>CI</th></tr>
<tr><td rowspan="3">Actor</td><td>0.3±0.02 | 0.3±0.02</td><td>10.83 | 10.55</td><td>1.32e-14 | 3.26e-14</td><td>1.532 | 1.492</td><td>[0.16, 0.23] | [0.15, 0.22]</td></tr>
<tr><td>0.74±0.01 | 074±0.01</td><td>17.25 | 17.08</td><td>0 | 0</td><td>2.439 | 2.415</td><td>[0.21, 0.27] | [0.21, 0.27]</td></tr>
<tr><td>0.23±0.01</td><td>11.39</td><td>2.22e-15</td><td>1.611</td><td>[0.1, 0.14]</td></tr>
<tr><td rowspan="3">Musician</td><td>0.3±0.01 | 0.3±0.01</td><td>13.24 | 12.84</td><td>< 0.001 | < 0.001</td><td>1.873 | 1.816</td><td>[0.16, 0.22] | [0.16, 0.22]</td></tr>
<tr><td>0.76±0.01 | 0.76±0.01</td><td>22.71 | 22.81</td><td>< 0.001 | < 0.001</td><td>3.211 | 3.225</td><td>[0.23, 0.28] | [0.24, 0.28]</td></tr>
<tr><td>0.3±0.01</td><td>15.12</td><td>< 0.001</td><td>2.139</td><td>[0.16, 0.21]</td></tr>
<tr><td rowspan="3">Politician</td><td>0.37±0.02 | 0.37±0.02</td><td>17.28 | 16.9</td><td>< 0.001 | < 0.001</td><td>2.443 | 2.39</td><td>[0.23, 0.29] | [0.23, 0.29]</td></tr>
<tr><td>0.8±0.01 | 0.8±0.01</td><td>27.01 | 26.86</td><td>< 0.001 | < 0.001</td><td>3.820 | 3.798</td><td>[0.28, 0.32] | [0.28, 0.32]</td></tr>
<tr><td>0.26±0.01</td><td>11.98</td><td>3.33e-16</td><td>1.694</td><td>[0.12, 0.17]</td></tr>
<tr><td rowspan="3">Apple</td><td>0.28±0.01 | 0.27±0.01</td><td>12.28 | 11.98</td><td>1.11e-16 | 3.33e-16</td><td>1.737 | 1.695</td><td>[0.14, 0.2] | [0.13, 0.19]</td></tr>
<tr><td>0.69±0.01 | 0.69±0.01</td><td>16.34 | 15.95</td><td>< 0.001 | < 0.001</td><td>2.311 | 2.256</td><td>[0.17, 0.21] | [0.16, 0.21]</td></tr>
<tr><td>0.27±0.02</td><td>10.24</td><td>9.06e-14</td><td>1.448</td><td>[0.13, 0.19]</td></tr>
<tr><td rowspan="3">Grape</td><td>0.35±0.02 | 0.35±0.02</td><td>11.27 | 10.72</td><td>3.33e-15 | 1.89e-14</td><td>1.593 | 1.516</td><td>[0.2, 0.29] | [0.19, 0.28]</td></tr>
<tr><td>0.75±0.02 | 0.75±0.02</td><td>16.33 | 15.95</td><td>< 0.001 | < 0.001</td><td>2.309 | 2.255</td><td>[0.22, 0.28] | [0.22, 0.28]</td></tr>
<tr><td>0.34±0.02</td><td>11.71</td><td>7.77e-16</td><td>1.656</td><td>[0.19, 0.27]</td></tr>
<tr><td rowspan="3">Pear</td><td>0.27±0.02 | 0.26±0.02</td><td>8.77 | 8.43</td><td>1.31e-11 | 4.27e-11</td><td>1.240 | 1.192</td><td>[0.12, 0.19] | [0.12, 0.19]</td></tr>
<tr><td>0.70±0.02 | 0.70±0.02</td><td>12.21 | 12.18</td><td>2.22e-16 | 2.22e-16</td><td>1.727 | 1.723</td><td>[0.17, 0.24] | [0.17, 0.24]</td></tr>
<tr><td>0.21±0.01</td><td>9.7</td><td>5.48e-13</td><td>1.372</td><td>[0.08, 0.12]</td></tr>
<tr><td rowspan="3">Beach</td><td>0.37±0.02 | 0.37±0.02</td><td>16.38 | 16.37</td><td>< 0.001 | < 0.001</td><td>2.317 | 2.315</td><td>[0.23, 0.3] | [0.23, 0.29]</td></tr>
<tr><td>0.77±0.01 | 0.77±0.01</td><td>24.19 | 23.86</td><td>< 0.001 | < 0.001</td><td>3.421 | 3.375</td><td>[0.25, 0.3] | [0.25, 0.3]</td></tr>
<tr><td>0.36±0.02</td><td>15.18</td><td>< 0.001</td><td>2.147</td><td>[0.22, 0.28]</td></tr>
<tr><td rowspan="3">Bridge</td><td>0.41±0.02 | 0.4±0.02</td><td>14.94 | 14.72</td><td>< 0.001 | < 0.001</td><td>2.113 | 2.082</td><td>[0.25, 0.33] | [0.25, 0.33]</td></tr>
<tr><td>0.8±0.01 | 0.8±0.01</td><td>24.62 | 25.03</td><td>< 0.001 | < 0.001</td><td>3.481 | 3.54</td><td>[0.28, 0.33] | [0.28, 0.33]</td></tr>
<tr><td>0.39±0.02</td><td>17.5</td><td>< 0.001</td><td>2.475</td><td>[0.25, 0.31]</td></tr>
</table>

Mountain	0.28±0.02 | 0.28±0.02<br>0.72±0.01 | 0.72±0.01<br>*0.18±0.01*	11.09 | 11<br>16.48 | 16.18<br>*6*	5.77e-15 | 7.88e-15<br>< 0.001 | < 0.001<br>*2.34e-07*	1.568 | 1.555<br>2.330 | 2.289<br>*0.849*	[0.14, 0.2] | [0.14, 0.2]<br>[0.19, 0.25] | [0.19, 0.25]<br>*[0.05, 0.1]*

For each class (category or subcategory), the statistics for the cross-validation (row 1: classifier accuracy, row 2: AUC) and central study decoding (row 3: classifier accuracy, *italicized*) are summarized in the mean ± SEM, *t*-value (DOF = 49) of one-sample T-tests (two-sided) with chance level (1/the number of operations for accuracy, 0.5 for AUC), *p*-value, effect size (Cohen's *d*), and 95% confidence interval in order. The cross-validation classifiers were validated with both individualized penalty (see Methods) and a single global penalty. The individualized penalty was applied on the classifiers that tested the central study data.

Supplementary Table 3. Summary statistics of the WM representation trajectories in the category-level (classifier) and item-level (RSA, Fig. 4). The data were evaluated for five 1.38 s (3 TR) time windows beginning at the onset of the manipulation instruction at 2.76 s after stimulus onset and extending to 9.66 s post-stimulus onset (window 1-5).

Neural decoding of a WM item	Category-level classifier				
	Window	T(49)	P	D	CI
Maintain vs. Replace-old	1	3.55	8.5e-04	0.503	[0.003, 0.03]
	2	5.95	3.53e-07	0.841	[0.01, 0.04]
	3	11.37	3.89e-15	1.609	[0.03, 0.06]
	4	14.05	< 0.001	1.99	[0.07, 0.11]
	5	12.23	5.55e-16	1.73	[0.11, 0.19]
Replace-new vs. Baseline	4	6.73	4.39e-08	0.951	[0.03, 0.09]
	5	10.37	2.98e-13	1.466	[0.09, 0.16]

For each paired T-test, (two-sided, FDR corrected) on the category-level classifier evidence value, *t*-value (DOF = 49), *p*-value, effect size (Cohen's *d*), and 95% confidence interval are summarized in order (Fig. 4A).

Removal of a WM item	Category-level classifier | Item-level RSA				
	Window	T(49)	P	D	CI
Replace	1	3.55 | -	8.5e-04 | -	0.503 | -	[0.01, 0.03] | -
	2	5.95 | -	3.53e-07 | -	0.841 | -	[0.02, 0.04] | -
	3	11.37 | 3.52	3.89e-15 | .002	1.609 | 0.498	[0.04, 0.05] | [0.01, 0.04]
	4	14.05 | 7.39	< 0.001 | 4.09e-09	1.99 | 1.046	[0.08, 0.1] | [0.04, 0.06]
	5	12.23 | 8.88	5.55e-16 | 4.43e-11	1.73 | 1.256	[0.13, 0.18] | [0.05, 0.08]
Suppress	2	- | 2.97	- | .008	- | 0.42	- | [0.01, 0.03]
	3	- | 3.63	- | .002	- | 0.513	- | [0.01, 0.03]
	4	3.97 | 3.56	0.001 | .002	0.562 | 0.503	[0.01, 0.03] | [0.01, 0.04]
	5	3.57 | -	0.002 | -	0.505	[0.01, 0.04] | -

Clear	2	2.96 | -	0.006 | -	0.419 | -	[0.003, 0.02]| -
	3	3.71 | 3.44	8.73e-04 | .002	0.525 | 0.486	[0.01, 0.03] | [0.01, 0.03]
	4	5.57 | 5.78	2.68e-06 | 2.56e-06	0.788 | 0.817	[0.02, 0.05] | [0.02, 0.05]
	5	7.12 | 5.44	4.29e-09 | 1.66e-06	1.007 | 0.77	[0.04, 0.08] | [0.03, 0.05]
Replace vs. Suppress	1	3.8 | -	3.95e-04 | -	0.538 | -	[0.01, 0.03] | -
	2	5.3 | -	3.47e-06 | -	0.749 | -	[0.01, 0.03] | -
	3	9.46 | -	2.04e-12 | -	1.338 | -	[0.03,0.05] | -
	4	10.92 | 4.09	2.5e-14 | 4e-04	1.544 | 0.579	[0.06, 0.08] | [0.01, 0.04]
	5	11.91 | 6.73	2.22e-15 | 1.76e-08	1.685 | 0.951	[0.1, 0.15] | [0.04, 0.07]
Replace vs. Clear	2	3.38 | -	0.002 | -	0.479 | -	[0.01, 0.03] | -
	3	6.77 | -	2.53e-08 | -	0.957 | -	[0.02, 0.04] | -
	4	9.94 | -	6.21e-13 | -	1.405 | -	[0.05, 0.07] | -
	5	10.81 | 3.77	7.11e-14 | 4.41e-04	1.529 | 0.533	[0.07, 0.11] | [0.01, 0.04]
Suppress vs. Clear	4	2.64 | -	2.77e-02 | -	0.373 | -	[0.003, 0.03]| -
	5	5.04 | 3.71	3.32e-05 | 5.35e-04	0.713 | 0.524	[0.02, 0.05] | [0.01, 0.05]

The one-sample T-test (vs. baseline = 0, two-sided, FDR corrected) results for the adjusted decoding value (removal – maintain; category-level | item-level) of each operation and the paired T-test (two-sided, FDR corrected) results across the operations are summarized in *t*-value, *p*-value, effect size (Cohen's *d*), and 95% confidence interval are summarized in order (Fig. 4B). Only the results from the significant windows are reported (- : n.s.).

Supplementary Table 4. Summary statistics of WM operation impact on the same-minus-different encoding fidelity.

Same-minus-different	M±SEM	T(49)	P	D	CI
Maintain	-0.03±0.01	2.96	.005 *	0.418	[0.01, 0.05]
Replace	-0.03±0.01	2.35	.023 *	0.332	[0.003, 0.05]
Suppress	0.04±0.01	4.41	5.66e-05 *	0.624	[0.02, 0.06]
Clear	-0.03±0.01	3.04	.004 *	0.429	[0.01, 0.06]
Maintain vs. Replace		0.34	.986	0.048	[-0.02, 0.03]
Maintain vs. Suppress		5.1	3.2e-05 *	0.721	[0.04, 0.1]
Maintain vs. Clear		0.17	.998	0.024	[-0.03, 0.03]
Replace vs. Suppress		4.73	1.11e-04 *	0.669	[0.04, 0.09]
Replace vs. Clear		0.46	.968	0.065	[-0.03, 0.04]
Suppress vs. Clear		5.37	1.27e-05 *	0.759	[0.05, 0.1]

Same- | Different-category	T(49)	P	D	CI
Maintain vs. Replace	1.94 | 2.37	.225 | .096	0.274 | 0.335	[-0.001, 0.05] | [0.003, 0.03]
Maintain vs. Suppress	5.95 | 0.68	1.68e-06 * | .905	0.841 | 0.096	[0.04, 0.09] | [-0.01, 0.02]
Maintain vs. Clear	0.21 | 0.003	0.997 | 1	0.03 | 0.0004	[-0.02, 0.03] | [-0.02, 0.02]
Replace vs. Suppress	4.16 | 2.36	7.22e-04 * | .098	0.588 | 0.334	[0.02, 0.06] | [0.004, 0.05]

Replace vs. Clear	2.07 | 1.71	0.177 | 0.326	0.293 | 0.243	[0.001, 0.05]	| [-0.003, 0.04]
Suppress vs. Clear	5.64 | 0.75	4.89e-06 * | 0.875	0.798 | 0.107	[0.04, 0.09]	| [-0.01, 0.02]

The one-sample T-test (vs. baseline = 0, two-sided) results for the encoding fidelity (same-minus-different) of each operation and the paired T-test (two-sided, Tukey-Kramer corrected) results for the three different encoding fidelities (same-minus-different, same-category, different-category) across the operations are summarized in *t*-value (DOF), *p*-value, effect size (Cohen's *d*), and 95% confidence interval in order (* denotes P < .05, Fig. 5).

Supplementary Table 5. The first contrast examined activation when the item needs to be manipulated (replace, suppress, clear) as compared to when no such manipulation was required (maintain). As in our prior study [2], this contrast mainly revealed activation in posterior regions of the brain, with relatively sparse activation in prefrontal cortex associated with cognitive control.

Region	BA	Max t	Voxel	X	Y	Z
Postcentral Gyrus (R)	BA2	6.56	12948	50	-24	50
Anterior Cingulate (L)	BA32	6.05	2442	-8	32	-12
Precuneus (L)	BA19	6.03	1129	-44	-76	42
Parahippocampal Gyrus (R)	BA28	6.87	872	20	-8	-14
Inferior Temporal Gyrus (R)	BA37	6.11	871	60	-60	-8
Cerebellum (R)	N/A	5.21	562	44	-72	-34
Transverse Temporal Gyrus (R)	BA41	5.23	535	64	-20	8
Middle Frontal Gyrus (L)	BA8	4.89	409	-26	30	44
Precuneus (R)	BA7	4.32	269	28	-60	32

Supplementary Table 6. The second contrast examined activation when an item had to be removed from working memory (suppress, clear) as compared to when an item remained in working memory (maintain, replace). Once again, the results were consistent with our prior report [2]**.** There was activation in several different regions involved in cognitive control, but unlike the prior contrast, these included many prefrontal regions, including anterior DLPFC.

Region	BA	Max t	Voxel	X	Y	Z
Middle Occipital Gyrus (R)	BA19	7.7	12316	38	-82	12
Insula (R)	BA13	9.3	3800	50	8	2
Superior Frontal Gyrus (R)	BA6	8.87	3247	10	6	72
Precentral Gyrus (L)	BA44	8.56	2479	-56	6	6
Supramarginal Gyrus (R)	BA40	8.01	1986	60	-36	36
Superior Frontal Gyrus (R)	BA9	6.95	1410	28	54	24
Cingulate Gyrus (L)	BA31	6.77	1344	-12	-24	42
Inferior Parietal Lobule (L)	BA40	8.56	1263	-62	-40	32
Superior Frontal Gyrus (L)	BA9	6.96	1105	-34	52	30

Supplementary Table 7. Finally, the remaining contrast examined differences when working memory had to be cleared of all thought (clear) as compared to when it did not (maintain, replace, suppress). As observed previously [2], there was significant more activation in the insula, as well as the SMA and frontopolar regions, than observed for the other conditions.

Region	BA	Max t	Voxel	X	Y	Z
Fusiform Gyrus (R)	BA19	8.07	6117	28	-58	-6
Precentral Gyrus (R)	BA44	8.5	4139	54	10	6
Middle Occipital Gyrus (L)	BA19	7.73	2276	-36	-84	10
Supramarginal Gyrus (R)	BA40	8.12	1656	56	-36	34
Superior Frontal Gyrus (R)	BA6	7.33	1273	12	6	70
Insula (L)	BA13	5.78	1115	-36	2	6
Fusiform Gyrus (L)	BA19	6.67	787	-26	-60	-6
Inferior Parietal Lobule (L)	BA40	5.99	654	-62	-40	40
Precentral Gyrus (R)	BA6	5.75	614	48	0	56
Superior Frontal Gyrus (L)	BA9	5.89	514	-32	54	30
Cerebellum (L)	N/A	5.65	383	-40	-44	-44

Supplementary Table 8. The contrast of suppress > clear revealed that the suppress condition revealed much more activation across a broad swath of lateral prefrontal cortex, consistent with our conclusion that the suppress condition is characterized by an active removal process (i.e., one that involves cognitive control). Alternatively, the contrast of clear > suppress revealed activation in the precuneus, as well as regions in the right hemisphere occipital regions adjacent to those that according to Neurosynth (https://neurosynth.org/) are significantly association with the term distraction. This finding is also consistent with our multivariate results suggesting that the clear condition works by removing information from the focus of attention in working memory, but that it does not necessarily influence the memory representation itself.

Region	BA	Max t	Voxel	X	Y	Z
Suppress > Clear						
Inferior Frontal Gyrus (L)	BA45	7.41	5400	-54	24	8
Superior Frontal Gyrus (L)	BA6	6.7	2587	-4	18	50
Lingual Gyrus (L)	BA17	5.8	1610	-10	-94	-4
Superior Temporal Gyrus (L)	BA39	5.31	1516	-56	-60	28
Middle Temporal Gyrus (L)	BA22	5.97	1139	-58	-40	4
Clear > Suppress						
Middle Occipital Gyrus (R)	BA37	4.37	393	44	-66	10
Middle Occipital Gyrus (L)	BA19	4.5	373	-42	-88	12
Precuneus (R)	BA7	4.35	285	6	-34	46
Precuneus (R)	BA7	4.07	259	26	-66	34

Supplementary Table 9. We also compared the suppress and maintain condition, which showed significantly different neural signature for the cognitive process but similar engagement in terms of holding representation in WM. The contrast of suppress > maintain showed greater activation in inferior frontal and opercular regions, which were involved in cognitive control. On the other hand, the contrast of maintain > suppress revealed activation in regions of the ventral visual stream suggesting that maintain requires holding specifics item details more than suppress.

Region	BA	Max t	Voxel	X	Y	Z
Suppress > Maintain						
Precuneus (L)	BA7	8.34	14609	-6	-74	40
Superior Frontal Gyrus (R)	BA6	8.06	4876	4	12	62
Superior Temporal Gyrus (L)	BA22	7.25	4719	-54	10	2
Superior Temporal Gyrus (R)	BA22	8.81	2059	54	14	0
Middle Frontal Gyrus (R)	BA6	6.53	689	44	2	50
Supramarginal Gyrus (L)	BA40	5.79	370	-54	-44	30
Caudate/Putamen	N/A	5.27	367	20	10	10
Superior Frontal Gyrus (R)	BA10	5.02	367	28	50	26
Cingulate Gyrus (L)	BA23	5.64	342	0	-20	32
Maintain > Suppress						
Cingulate Gyrus (R)	BA31	6.07	2988	6	-38	40
Parahippocampal Gyrus (L)	BA36	6.97	2883	-36	-24	-18
Inferior Temporal Gyrus (R)	BA37	6.66	2281	60	-56	-8
Insula (L)	BA13	5.88	2182	-36	-8	16
Medial Frontal Gyrus (L)	BA11	7.19	1684	-8	32	-12
Superior Parietal Lobule (R)	BA7	7.55	999	38	-74	48
Middle Temporal Gyrus (L)	BA21	5.17	682	-66	-24	-12
Precuneus (L)	BA19	7.07	588	-40	-80	38
Middle Frontal Gyrus (L)	BA8	5.91	380	-18	24	42
Posterior Cingulate	BA23/31	4.6	254	12	-52	24

Supplementary References

1. Klein, A. & Tourville, J. 101 Labeled Brain Images and a Consistent Human Cortical Labeling Protocol. *Front. Neurosci.* **6**, (2012).
2. Banich, M. T., Mackiewicz Seghete, K. L., Depue, B. E. & Burgess, G. C. Multiple modes of clearing one's mind of current thoughts: Overlapping and distinct neural systems. *Neuropsychologia* **69**, 105–117 (2015).
